# Phytochemical Profiles and Antioxidant and Antimicrobial Activities of the Leaves of *Zanthoxylum bungeanum*

**DOI:** 10.1155/2014/181072

**Published:** 2014-07-24

**Authors:** Yujuan Zhang, Ziwen Luo, Dongmei Wang, Fengyuan He, Dengwu Li

**Affiliations:** College of Forestry, Northwest A&F University, Yangling 712100, China

## Abstract

The ethanol crude extracts (ECE) and their subfractions from *Zanthoxylum bungeanum* leaves were prepared and their phytochemical profiles and antioxidant and antimicrobial activities were investigated. Moreover, the effective HPLC procedure for simultaneous quantification of twelve compounds in *Z. bungeanum* leaves was established. The correlation between the phytochemicals and antioxidant activity was also discussed. The ethyl acetate fraction (EAF) had the highest total phenolic (97.29 mmol GAE/100 g) and flavonoid content (67.93 mmol QE/100 g), while the greatest total alkaloid content (4.39 mmol GAE/100 g) was observed in the chloroform fraction (CF). Twelve compounds were quantified by RP-HPLC assay. EAF exhibited the highest content of quercitrin, kaempferol-3-rhamnoside, quercetin, sesamin, and nitidine chloride (125.21, 54.95, 24.36, 26.24, and 0.20 mg/g); acetone fraction (AF) contained the highest content of chlorogenic acid, rutin, hyperoside, and trifolin (5.87, 29.94, 98.33, and 31.24 mg/g), while kaempferol-3-rhamnoside, xanthyletin, and sesamin were rich in CF. EAF and AF exhibited significant DPPH, ABTS radical scavenging abilities and reducing power (FRAP), whereas CF exhibited significant antifungal activity. Moreover, EAF also showed stronger antibacterial activity. In conclusion, *Z. bungeanum* leaves have health benefits when consumed and could be served as an accessible source for production of functional food ingredients and medicinal exploration.

## 1. Introduction

*Zanthoxylum bungeanum*, known as the Da Hongpao Huajiao, which belongs to the* Zanthoxylum* genus of the family Rutaceae, is widely distributed in Hebei, Shanxi, Sichuan, Gansu, and Shandong provinces of China and some Southern Asian countries [1]. Just like other species of this genus,* Z. bungeanum* has a distinctive tingling taste. Due to its fresh aroma and taste, the dried fruits are used ground or whole as a spice in local cuisines, which can stimulate saliva production and increase appetite [2]. Consisting of salt and Sichuan pepper (*Z. bungeanum*), hua jiao yen is often used as a condiment in barbecue foods, such as chicken tikka or roast duck. Apart from its common application as a condiment to make foods more flavoring, each part of* Z. bungeanum* has numerous medicinal virtues. In traditional Chinese medicine, the pericarp can be used for gastralgia and dyspepsia; the seed is reported to be antiphlogistic and diuretic; the leaves are considered carminative, stimulant, and sudorific; the root can cure epigastric pains and treat bruises, eczema, and snakebites [3–7]. Recent experimental studies have shown that the pericarp of* Z. bungeanum* possesses cardiovascular activity [8]; it also can be used as an ingredient in cosmetic products [9]; methanol extracts of* Z. bungeanum* have anti-inflammatory activity [10]; the essential oil of seed and fruit exhibits marked antioxidant activity as well as antimicrobial activity [11–13].

The leaves of* Z. bungeanum* are edible; they taste acrid and innocuous. In some rural areas, local people eat the new leaves as vegetables in spring seasons [14]. Furthermore, it is also commonly used as condiments in Chinese cuisine and in the preparation of refreshments to add flavor [11]. In spite of its long history of consumption, only a few people pay attention to the chemical work on this material. Fan and coworkers did a study on the ultrasonic-assisted extraction of total flavonoids from* Z. bungeanum* leaves [15]. Yang and coworkers identified 13 polyphenolics from the leaves of* Z. bungeanum* grown in Hebei, China, by HPLC/MS, among which chlorogenic acid, hyperoside, and quercitrin were the major constituents [1]. But, there are still some unclear points for consumers on the phytochemical profiles and physiological effects of this edible material. In order to fully investigate, utilize, and develop this material, we designed an experiment: (1) to evaluate the contents of total flavonoid, phenol, and alkaloid in the different polarity fractions of* Z. bungeanum *leaves; (2) to measure the antioxidant and antimicrobial activities of the different polarity fractions; (3) to quantify the content of twelve natural compounds (chlorogenic acid, epicatechin, rutin, hyperoside, trifolin, quercitrin, kaempferol-3-rhamnoside, quercetin, nitidine chloride, chelerythrine, xanthyletin, and sesamin) in the different polarity fractions by RP-HPLC analysis; and (4) to compare the similarities and differences of the phytochemical composition in the different polarity fractions. Based on these results, the most bioactive fraction could be selected as a potential source of natural antioxidants and antiseptics. In addition, phytochemicals might be responsible for their profound bioproperties which will be screened out. The results also could explain its frequent addition to the Chinese diet for promoting human health and for disease prevention.

## 2. Materials and Methods

### 2.1. Plant Material and Chemicals

*Z. bungeanum* leaves were collected from Taibai Mountains of Shaanxi province, China, in September, 2012, and authenticated by the Herbarium of the Northwest A&F University, Yangling, China.

The following were obtained: Folin-Ciocalteu reagent (Shanghai Solarbio Bioscience & Technology Co., Ltd., China); 1,1-diphenyl-2-picrylhydrazyl (DPPH), 2,2-azino-bis(3-ethylbenzothiazoline-6-sulphonic acid) diammonium salt (ABTS), 2,4,6-tripyridyl-s-triazine (TPTZ), and 6-hydroxy-2,5,7,8-tetramethylchroman-2-carboxylic acid (Trolox) (Sigma-Aldrich Co., St. Louis, USA); vanillin, bromocresol green, tetrahydrofuran (THF), sodium borohydride, and trifluoroacetic acid (Chengdu Kelong Chemical Co., Ltd., China); chloranil (Aladdin Industrial Corporation, Shanghai, China); gallic acid, chlorogenic acid, epicatechin, rutin, hyperoside, trifolin, quercitrin, kaempferol-3-rhamnoside, quercetin, nitidine chloride, chelerythrine, xanthyletin, and sesamin (Shanghai Winherb Medical Science Co., Ltd.); and amphotericin and benzylpenicillin (Shanghai Sunny Biotechnology Co. Ltd., China). All solvents used were of AR-grade. Deionized water (18 MΩ cm) was used to prepare aqueous solutions.

Twenty fungi (*Botrytis cinerea, Piricularia oryzae, Physalospora piricola, Glomerella cingulata,* and* Venturia pyrina*, etc.) and two Gram-positive (*Staphylococcus aureus and Bacillus subtilis*) and one Gram-negative (*Escherichia coli*) bacteria were provided by the College of Resources and Environment, Northwest A&F University, China.

### 2.2. Preparation of the Ethanol Crude Extracts and Fractions

The air-dried and powdered leaves of* Z. bungeanum *(9.40 Kg) were extracted using 95% ethanol at room temperature for 24 h, with solid to liquid ratio of 1 : 5, repeating 6 times. The ethanol crude extracts (ECE, 1839.96 g) were filtered and evaporated to dryness by rotary evaporation at 45°C under reduced pressure. 15.56 g of ECE was stored for further analysis. The remaining ECE (1824.40 g) was further fractioned by column chromatography on silica gel (silica gel 200–300 mesh, 120∗10 cm i.d., flow rate 10 mL/min), successively eluting with petroleum ether, chloroform, ethyl acetate, acetone, and methanol. The eluents of the five different polarity solvents were collected separately and evaporated to dryness by rotary evaporation at 45°C under reduced pressure. Thus, the different polarity fractions (PEF, 105.98 g; CF, 112.76 g; EAF, 40.70 g; AF, 124.93 g; and MF, 624.35 g) were obtained and carefully stored at −20°C and protected from light until further analysis [16, 17].

### 2.3. Determination of Total Flavonoid Content (SBC Method)

The total flavonoid content (TFC) was determined based on a SBC assay using sodium borohydride/chloranil as described previously [18–20]. This assay allows the detection of numerous flavonoid varieties, including flavones, flavonols, flavonones, flavononols, isoflavonoids, and anthocyanins [20]. A calibration curve was constructed to create a standard using different concentrations of quercetin (0.1–10.0 mM). TFC of extracts and different polarity fractions from* Z. bungeanum *leaves were expressed as mmol quercetin equivalent per 100 g and all samples were evaluated in triplicate.

### 2.4. Determination of Total Phenolic Content

The total phenolic content (TPC) was determined using the Folin-Ciocalteu colorimetric method as described previously [21, 22]. TPC was calculated by gallic acid equivalent from the calibration curve from the gallic acid standard solutions (20–300 *μ*g/mL). TPC of extracts and different polarity fractions from* Z. bungeanum *leaves were expressed as mmol gallic acid equivalent per 100 g, and all the samples were measured in triplicate.

### 2.5. Determination of Total Alkaloid Content

The total alkaloid content (TAC) was determined using the acid dye colorimetric method with the following modifications [23]. Chelerythrine (0.2–1.0 mg/mL) was used as a reference for the calibration curve. TAC of extracts and different polarity fractions from* Z. bungeanum *leaves were expressed as mmol of chelerythrine equivalent per 100 g, and all of the samples were analyzed in triplicate.

### 2.6. Assessment of the Twelve Compounds by HPLC

The content of twelve compounds (chlorogenic acid, epicatechin, rutin, hyperoside, trifolin, quercitrin, kaempferol-3-rhamnoside, quercetin, nitidine chloride, chelerythrine, xanthyletin, and sesamin) was assayed using an Agilent Technologies 1260 series liquid chromatograph (RP-HPLC) coupled with a variable wavelength detector. The quantification was carried out on a SB-C18 reversed phase column (5 *μ*m, 4.6∗250 mm) at ambient temperature [24, 25]. The mobile phase consisted of water with 0.5% trifluoroacetic acid (solvent A) and acetonitrile with 0.5% trifluoroacetic acid (solvent B). The flow rate was 0.8 mL/min. The gradient program was set as follows: from 0 to 30 min, eluent B was increased from 15% to 35%; from 30 to 35 min, eluent B was increased from 35% to 65%; and from 35 to 55 min, eluent B was increased from 65% to 100% and then maintained at 100% for 10–20 min. The injection volume was 20 *μ*L and the detection wavelength was 254 nm. Samples were filtered through a 0.22 *μ*m membrane filter prior to injection. The major constituents in the ECE and its five different polarity fractions were identified by comparing their retention times and the spectral characteristics of their peaks with those of the standards. The analyses were all performed in triplicate.

### 2.7. Validation of the HPLC Method

The linear calibration curves contained six different concentrations of each standard compound by a series of appropriate dilutions with mobile phase. All calibration curves were constructed by plotting the peak areas of the standard substances versus the corresponding concentrations of the injected standard solutions.

The HPLC procedure was also validated for its precision, reproducibility, and recovery test [26]. To determine the precision of the procedure, the standard compounds solutions were analyzed in triplicate for three times within one day, while for interday variability, the samples were examined in triplicate for three consecutive days. To determine the reproducibility, six working solutions were prepared using the ethanol crude extractions (ECE, 5 mg/mL). The recovery test was used to evaluate the accuracy of the proposed method. Accuracy was determined by adding different concentrations of the mixed standard solutions into the known amounts of sample solutions of ECE. Then the compounds in the resultant samples were analyzed with the proposed method. The recovery was calculated as follows:

(1) Recovery(%)   =  (total  detected  amount−original  amountadded  amount)∗100.

The RSD values were taken as measurements for precision, reproducibility, and recovery tests.

### 2.8. DPPH Radical Scavenging Activity

DPPH radical scavenging activity was evaluated using the method described by Yen and Chen [27] and Sultana et al. [28] with some modifications [17]. A 2 mL volume of the sample solutions (20–1000 *μ*g/mL) or the positive controls rutin and quercetin (1–200 *μ*g/mL) was added to 2 mL of DPPH solution (100 *μ*M); and the absorbance was measured with a spectrophotometer (Shimadzu UV-1800) at 517 nm after standing in the dark for 30 min. All the tests and the controls were repeated in triplicate. The DPPH free radical scavenging activity was calculated using the following equation:

(2) $$\begin{array}{c} \text{Scavenging}\left(\%\right)=\left[\frac{1-\left(A_{i}-A_{j}\right)}{A_{o}}\right]\times 100\%, \end{array}$$

where *A*_*o*_ is the absorbance of ethanol (2 mL) and DPPH*·* (2 mL), *A*_*i*_ is the absorbance of the tested sample (2 mL sample and 2 mL DPPH*·*), and *A*_*j*_ is the absorbance of the blank (2 mL sample and 2 mL ethanol).

### 2.9. ABTS Radical Cation Decolorization Assay

Antioxidant activity was determined according to the decolorizing free radical ABTS*·* ^+^ method [29] as described previously [30–32]. For each analysis, 100 *μ*L of sample (1 mg/mL) and the positive controls (rutin and quercetin, 0.05 mg/mL) was added to 3.9 mL of the ABTS*·* ^+^ solution, and the decrease in absorbance at 734 nm was recorded within 6 min. The results were expressed as micromoles of trolox equivalent per g. All determinations were carried out in triplicate.

### 2.10. Ferric Reducing Antioxidant Power (FRAP) Assay

The FRAP assay [33] was performed with some modifications [31]. For each analysis, 400 *μ*L of the sample (1 mg/mL) and the positive controls (rutin and quercetin, 0.05 mg/mL) was added to 3 mL of the FRAP solution. The increase in absorbance at 593 nm was recorded in 15 s intervals over the course of 30 min at 37°C. The FRAP results were expressed as micromoles of trolox equivalent per g. All determinations were carried out in triplicate.

### 2.11. Antifungal Activity

Antifungal assays [34] were performed with some modifications as described by Ai et al. [35], Wang et al. [17], Hsu et al. [36], and Tian et al. [37]. Each extract and fraction was dissolved in different proportions of acetone and water, that is, 100% acetone for PEF, CF, EAF, and AF and 50% acetone for ECE and MF. The treated dishes were incubated in the dark at 27.5–28.5°C for 72 h at moderate humidity. The relative growth inhibition (%) of the test sample compared with the control was calculated as follows:

(3) $$\begin{array}{c} \text{Inhibitory}\;\text{activity}\left(\%\right)=\left[\frac{\left(C-T\right)}{\left(C-4\;\text{mm}\right)}\;\right]\times 100\%, \end{array}$$

where *C* is the colony diameter of the mycelium on the control plate (mm) and *T* is the colony diameter of the mycelium on the test petri plate (mm).

*B. cinerea*,* P. oryzae*,* P. piricola*,* G. cingulata*, and* V. pyrina* were chosen for growth kinetics assays. The solutions of ECE, PEF, CF, EAF, AF, and MF were serially diluted by the twofold serial dilution method and added to PDA with concentrations ranging from 6.25 to 100 mg/mL. Amphotericin was used as standard. And the concentration of the sample required for 50% inhibitory activity (EC_50_) was calculated using linear regression analysis. All experiments were conducted in triplicate.

### 2.12. Antibacterial Activity

The paper disc diffusion method, also known as the agar diffusion method, was used to detect the antibacterial activity of the extracts and fractions of the leaves of* Z. bungeanum *[38, 39]. The beef extract peptone medium was inoculated with 3 *μ*L aliquots of culture containing approximately 10^5^ cfu/mL of each organism. Sterilized filter paper discs (5 mm) were soaked in 5 mL of various concentrations (6.25 to 100 mg/mL) of samples. Benzylpenicillin was used as standard (0.01 to 10 mg/mL). The paper discs soaked in the solvent without extracts or fractions (80% acetone) served as black control. The MIC values were determined as the lowest concentration of extracts inhibiting visible growth of each organism on the agar plate. The soaked discs were placed in the plates and incubated for 24 h at 28°C. Following the incubation period, the inhibition zones formed in the medium were measured in millimeters (mm). All the tests were performed in triplicate and the MIC values were calculated.

### 2.13. Statistical Analysis

All results were expressed as the mean ± standard deviation (SD). The significant difference was calculated by SPSS one-way ANOVA followed by Duncan's test; values <0.05 were considered to be significant (SPSS Inc., Chicago). The linear correlations among the various parameters were also investigated using the SPSS 18.0 software.

## 3. Results and Discussion

### 3.1. Total Phenolic, Flavonoid, and Alkaloid Content

The total phenolic, flavonoid, and alkaloid content of ethanol crude extracts (ECE) and its five different polarity fractions (PEF, CF, EAF, AF, and MF) from* Z. bungeanum *leaves were screened and compared. According to the results presented in Table 1, there was a statistically significant difference (*P* < 0.05) among all the samples investigated. EAF exhibited the highest TFC (67.93 mmol/100 g) and TPC (97.29 mmol/100 g) followed by AF (47.62 mmol/100 g for TFC; 62.87 mmol/100 g for TPC), which were much higher than ECE and other fractions. Compared with the high flavonoid and phenolic content, alkaloid yields were the lowest, since this group of compounds is sparsely distributed and more specific of genera and species [40]. Among the extracts and fractions of* Z. bungeanum *leaves, CF exhibited the greatest TAC (4.39 mmol/100 g) followed by PEF (1.71 mmol/100 g). Chen et al. reported that 23 alkaloids were isolated from the CHCl_3_ and MeOH extracts of the root bark of* Z. simulans* [41]. Ren and Xie reported 6 alkaloids from the root of* Z. bungeanum* [42]. We can hypothesize that CF from the leaves of* Z. bungeanum *can be further fractioned to gain bioactive alkaloids.

### 3.2. HPLC Analysis of the Extracts and Fractions

As seen in Table 2, the linear regression results indicated good linear correlation by the correlation coefficients between 0.9991 and 0.9999 for all of the standard compounds within the appropriate concentration ranges. The precision of the analytical method was analyzed in triplicate for three times within one day, while for interday variability, the samples were examined in triplicate for three consecutive days, and the RSDs of the peak areas were estimated to be 0.71–1.51% (*n* = 6). The repeatability of the method was determined by injecting the ECE for six times, while the peak area of the twelve detected compounds was recorded, and the RSDs of their peak area varied from 0.16 to 2.94%. To confirm the accuracy of the method, a recovery experiment was performed by mixing quantified samples with specific quantities of standard compounds. The average percentages of recovery of the twelve standard compounds ranged from 98.37 to 103.76%. In addition, the RSDs varied from 0.35 to 1.66% (*n* = 6). All the results demonstrated that the conditions of the analysis were repeatable and accurate.

The content of twelve compounds in the extracts and its five fractions from* Z. bungeanum *leaves were determined by matching their retention times against those of the standards (Table 3). Good correlation was observed between the peak area and the content. We established a standard HPLC method to determine 12 phytochemicals from the leaves of* Z. bungeanum *simultaneously. Epicatechin (27.45 mg/g), rutin (16.86 mg/g), hyperoside (19.25 mg/g), quercitrin (16.73 mg/g), chlorogenic acid (3.78 mg/g), kaempferol-3-rhamnoside (3.75 mg/g), trifolin (4.53 mg/g), and sesamin (5.13 mg/g) were the major phenolic components in ECE, other compounds (quercetin, nitidine chloride, chelerythrine, and xanthyletin) had a lower level of content (less than 1 mg/g). Among the 5 subfractions, the EAF exhibited the highest content of quercitrin (125.21 mg/g), kaempferol-3-rhamnoside (54.95 mg/g), quercetin (24.36 mg/g), nitidine chloride (0.20 mg/g), and sesamin (26.24 mg/g). The AF exhibited the highest content of chlorogenic acid (5.87 mg/g), rutin (29.94 mg/g), hyperoside (98.33 mg/g), and trifolin (31.24 mg/g). The CF exhibited the highest content of xanthyletin (0.09 mg/g), while the MF had the highest content of epicatechin (39.32 mg/g) and chelerythrine (0.09 mg/g). It is indicated that the major phytochemicals, especially phenolic compounds, were concentrated in EAF and AF, which may result from the enrichment effects during chromatographic fractionation. And we hypothesized that these bioactive phytochemicals might be responsible for their profound bioproperties.

**Figure 1:**
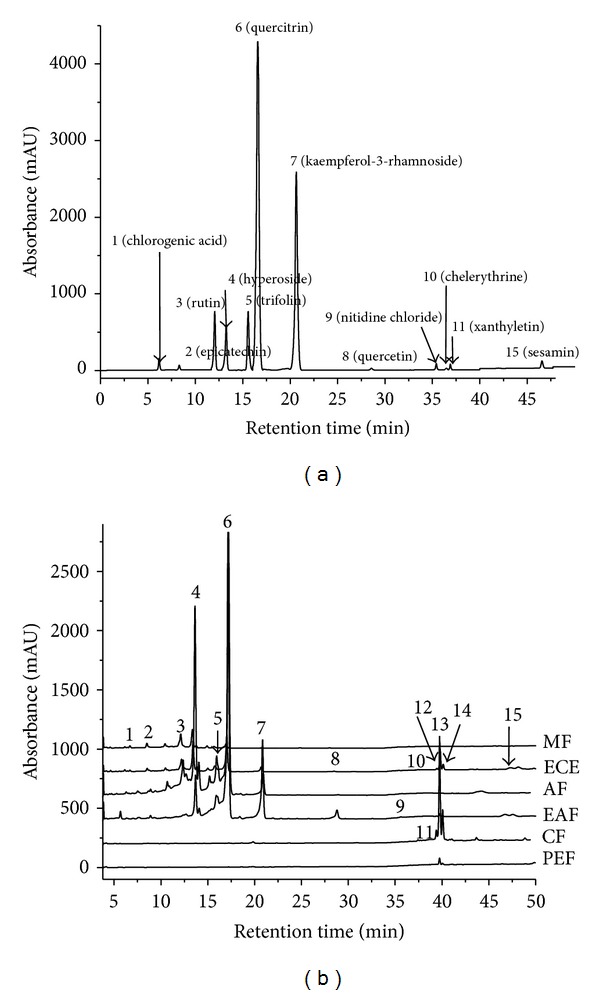
HPLC analysis of extracts and fractions from* Z. bungeanum* leaves. (a) Chromatography of the twelve standard compounds. (b) Chromatography of the ethanol extracts and their five fractions (ECE, PEF, CF, EAF, AF, and MF) monitored at 254 nm and identified by their retention time (min): chlorogenic acid (6.62, peak 1), epicatechin (8.31, peak 2), rutin (12.12, peak 3), hyperoside (13.61, peak 4), trifolin (15.73, peak 5), quercitrin (16.82, peak 6), Kaempferol-3-rhamnoside (20.61, peak 7), quercetin (28.32, peak 8), nitidine chloride (35.61, peak 9), chelerythrine (36.95, peak 10), xanthyletin (37.20, peak 11), and sesamin (46.44, peak 15). Peaks 9–11 were very weak.

Chromatography of the ECE and its five different polarity fractions revealed that there are significant differences among the tested samples (Figure 1(b)).  Figure 1(a) was the HPLC chromatograms of the 12 standard compounds. ECE contained all the phytochemicals detected and exhibited the richest peaks. But further fractionation of ECE by column chromatography on silica gel produced five subfractions with even higher content of all the detected phytochemicals. Among the five subfractions, peaks 1–9 (chlorogenic acid, epicatechin, rutin, hyperoside, trifolin, quercitrin, kaempferol-3-rhamnoside, quercetin, and nitidine chloride) were common peaks in EAF and AF. Peak 7 (kaempferol-3-rhamnoside), peak 11 (xanthyletin), and peaks 12–15 were common peaks detected in PEF and CF. The peak area of peaks 11–14 was the greatest in CF. Finally, peaks 1–6 (chlorogenic acid, epicatechin, rutin, hyperoside, trifolin, and quercitrin) and peak 10 (chelerythrine) were detected in MF at low levels, except for peak 2.

The present results exhibited significant differences with the reported study on the phytochemical composition of the* Z. bungeanum *leaves of Hebei, China [1]. Three major compounds (trifolin, kaempferol-3-rhamnoside, and quercetin) detected in the* Z. bungeanum *leaves of Taibai were not identified in that of Hebei. Nevertheless, other major phytochemicals, such as chlorogenic acid, epicatechin, rutin, hyperoside, and quercitrin, were both detected in the* Z. bungeanum *leaves of Taibai and in that of Hebei, though the contents were significantly different. The main reasons for these variations may be some geographical differences and gene mutation [43, 44]. Further isolation and purification of the fractions (EAF, AF, and CF) with the richest phytochemicals ought to be conducted, and later chemical structures of the new bioactive compounds need to be analyzed.

### 3.3. DPPH Radical Scavenging Activity

**Figure 2:**
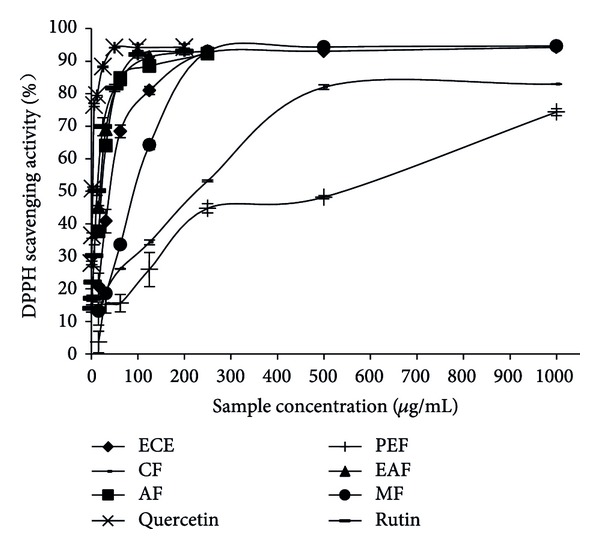
Scavenging effect on DPPH radical of extracts and fractions from* Z. bungeanum* leaves. Rutin and quercetin were used as the positive controls.

In the present study, the ethanol crude extracts (ECE) and its five different polarity fractions showed DPPH radical scavenging activity in a dose dependent manner at concentration of 20–1000 *μ*g/mL (Figure 2). The EAF and AF exhibited higher DPPH radical scavenging activity than ECE and other fractions. In order to further quantify the DPPH radical scavenging activity, the IC_50_ values of the extracts and five fractions were determined and shown in Table 1. All the extracts and fractions showed significant (*P* < 0.05) differences in their ability to reduce the DPPH radical; EAF and AF were selected as the most effective fractions with the highest DPPH radical scavenging ability, which were not significantly different with the reference compound (rutin, IC_50_ =10.42 *μ*g/mL). As seen in Table 1, the IC_50_ value of EAF was 13.20 *μ*g/mL, which was not significantly different from that of AF (18.55 *μ*g/mL) but was 3.1-, 6.5-, 12.8-, and 28.6-fold lower than that of ECE, MF, CF, and PEF, respectively (*P* < 0.05). Because the antioxidant activities were inversely correlated with the IC_50_ values, the DPPH radical-scavenging activity, in decreasing order, was EAF > AF > MF > CF> PEF.

### 3.4. ABTS Radical Cation Decolorization Activity

Another effective method to measure radical scavenging activity is the ABTS radical cation decolorization assay, which showed similar results to those obtained in the DPPH reaction. The scavenging activity of the extracts on free radical ABTS generated by potassium persulfate was compared with a standard amount of trolox. The result was calculated as micromoles of trolox equivalent per g. The ABTS radical scavenging ability of the extracts and fractions from* Z. bungeanum *leaves compared to rutin and quercetin has been depicted in Table 1; we also found that EAF and AF were the most effective fractions with the highest ABTS radical scavenging abilities. The ABTS radical scavenging ability of EAF was 2147.83 *μ*mol Trolox/g, which was not significantly different from that of AF (2044.58 *μ*mol Trolox/g, *P* < 0.05) but was 1.9-, 2.9-, 3.8-, and 8.1-fold higher than that of ECE, MF, CF, and PEF, respectively (*P* < 0.05).

### 3.5. Ferric Reducing Antioxidant Power (FRAP)

The FRAP values of the extracts and fractions from* Z. bungeanum* leaves have been depicted in Table 1. EAF and AF were also screened as the most effective fractions with the highest reducing values, which were rather consistent with the results of the scavenging capacity on DPPH and ABTS radical. It is exhibited that the reducing ability of EAF was 615.88 *μ*mol Trolox/g, which was not significantly different from that of AF (594.15 *μ*mol Trolox/g, *P* < 0.05) but was 1.9-, 3.2-, 3.6-, and 7.6-fold higher (*P* < 0.05) than that of ECE, MF, CF, and PEF, respectively.

### 3.6. Correlation between the Total Phenolic and Flavonoid Content and the Antioxidant Assays

Based on the correlation matrix (Table 4), each coefficient was assessed to establish the correlations between different assays. As displayed in Table 4, a high correlation was observed among the three methods for antioxidant activity measurement (−0.777 ≤ *r* ≤ 0.999, *P* < 0.01), indicating a great degree of equivalence among the measurements. Okonogi et al. demonstrated that the relationship between ABTS radical scavenging activities and the DPPH_IC50_ of the samples was nonlinear (*r* = −0.797). However, the logarithmic values of DPPH_IC50_ against ABTS radical scavenging activities gave good linearity (*r* = −0.968) [45]. Thus, the ABTS result was in good agreement with that of the DPPH assay; among the evaluated extracts and fractions, EAF and AF were selected as two fractions with the highest free radical and hydroxyl radical-scavenging activities. The FRAP values exhibited a significant linear correlation with ABTS result (*r* = 0.999, *P* < 0.01) and log values of DPPH_IC50_ (*r* = −0.958, *P* < 0.01), indicating that the phytochemicals with radical scavenging abilities also possessed reducing abilities. Log DPPH_IC50_, ABTS, and FRAP results were significantly correlated with the TFC (*r* = −0.922 for log DPPH_IC50_, *P* < 0.01; *r* = 0.924 for ABTS, *P* < 0.01; *r* = 0.923 for FRAP, *P* < 0.01). According to Prior and others [46] and Huang and others [47], the Folin-Ciocalteu method (used for determination of the total phenolic content) is based on oxidation-reduction reactions (single electron transfer (SET)) and can thus be considered as one of the methods for the determination of antioxidant activity. In addition, in our present study, good correlations were also observed between the TPC and log DPPH_IC50_ or ABTS results (*r* = −0.916 for log DPPH_IC50_, *P* < 0.05; *r* = 0.827 for ABTS, *P* < 0.05).

In our present study, it can be inferred that EAF and AF contained the highest total polyphenol and flavonoid levels and exhibited the highest antioxidant capacity among the five different polarity fractions. Since the antioxidant activity of plants depend on the amount and type of phenolic compounds that occur in them [48], we hypothesized that the phenolic compounds of the analyzed extracts and fractions were responsible for the profound antioxidant effects. Due to the enrichment effects during the chromatography fractionation, EAF and AF were effective in the recuperation of compounds with good reducing capacity and good electron donors.

### 3.7. Antifungal Activity

The antifungal activity of ECE, PEF, CF, EAF, AF, and MF against 20 varieties of plant pathogenic fungi was studied using the mycelial growth method. The inhibition of ECE ranged between 6.00 and 65.22%, while those of PEF, CF, EAF, AF, and MF were between 10.00 and 70.00% at a concentration of 50 mg/mL. Five plant pathogenic fungi (*B. cinerea*,* P. oryzae*,* P. piricola*,* G. cingulata, *and* V. pyrina*) with higher antifungal activity (over 50%) were chosen for further growth kinetics assays (Table 5).

**Figure 3:**
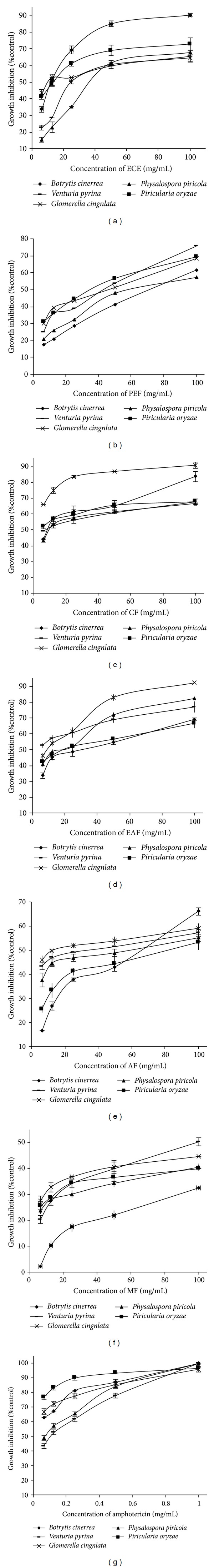
Inhibitory activity of extracts and fractions from* Z. bungeanum *leaves against 5 plant pathogenic fungi. Amphotericin was used as the positive control.

The antifungal kinetics of extracts and fractions of* Z. bungeanum *leaves were tested on the five selected fungi. The phytochemicals of* Z. bungeanum *leaves inhibited fungal growth (2.32–92.10%) at concentrations of 6.25–100 mg/mL (Figure 3). The growth inhibition of each sample increased with concentration and then plateaued, notwithstanding the increases in concentration. At 100 mg/mL concentrations of CF and EAF, the inhibitory activity of* G. cingulata* was 90.79% and 92.10%, respectively (Figures 3(c) and 3(d)). At 50 mg/mL concentrations, significant inhibitory activity (above 50%) was also observed for ECE, PEF, CF, EAF, and AF (Figures 3(a), 3(b), 3(c), 3(d), and 3(e)), indicating that the phytochemicals of* Z. bungeanum *leaves possessed broad-spectrum antifungal property; yet the MF (Figure 3(f)) exhibited less inhibitory activity (less than 50%) than the five selected pathogenic fungi. Amphotericin was used as the positive control (Figure 3(g)).

As seen in Table 6, the CF, with the lowest EC_50_ values of 0.83, 9.39, 4.18, 10.89, and 5.35 mg/mL against the growth of* G. cingulata*,* B. cinerea*,* P. oryzae*,* P. piricola*, and* V. pyrina* separately, exhibited the greatest inhibitory activity closely followed by EAF, with EC_50_ values of 9.25, 24.39, 17.81, 13.73, and 8.11 mg/mL, respectively (*P* < 0.05). CF and EAF exhibited greater antifungal activities, which might be further studied to determine whether this activity can be retained* in vivo*. The fractions (CF and EAF) with low and medium polarity phytochemicals exhibited the highest antifungal activities. These results have shown that CF and EAF from* Z. bungeanum* leaves might be an attractive alternative for the use of a natural product for control of fungi that attack food and crops, avoiding fungicides application.

### 3.8. Antibacterial Activity

MIC values of extracts and fractions of* Z. bungeanum* leaves were shown in Table 7. The control (80% acetone) did not inhibit any of microorganisms tested. The EAF showed the best antibacterial activity against both Gram-positive and Gram-negative bacteria,* S. aureus *(2.38 mg/mL),* E. coli* (2.32 mg/mL), and* B. subtilis* (4.24 mg/mL), and were not significantly different from those of MF (2.65, 3.10, and 4.10 mg/mL, resp.). Benzylpenicillin was only effective in the inhibition of Gram-positive bacteria. With the rapid emergence of multiple drug resistant pathogenic strains and the adverse side effects due to the use of conventional antibiotics, the discovery of new antimicrobial agents is a vital aspect of research and development in the realm of public health [49]. It is promising that EAF from* Z. bungeanum* leaves may harbor therapeutic compounds with significant antibacterial activity.

## 4. Conclusions

Phytochemical profiles and bioactivities of extracts and fractions from the leaves of* Z. bungeanum *were studied. Based on our results, EAF, AF, and CF were selected as the most effective fractions due to higher phytochemical contents and significant bioactivities. Moreover, a simple, rapid, and effective HPLC procedure for simultaneous quantification of twelve compounds in* Z. bungeanum* leaves was established. Our current work has shown that the phytochemicals present in* Z. bungeanum* leaves have potent bioproperties and that the antioxidant properties are positively correlated with the total flavonoid and phenolic content. HPLC analysis indicated that the major phytochemicals (chlorogenic acid, epicatechin, rutin, hyperoside, trifolin, quercitrin, kaempferol-3-rhamnoside, quercetin, sesamin, and nitidine chloride) were concentrated in the EAF and AF, which may be due to the enrichment effects during chromatographic fractionation. These bioactive phytochemicals might be responsible for their profound bioproperties. Furthermore, some lower polarity phytochemicals, such as kaempferol-3-rhamnoside, xanthyletin, sesamin, and other unknown compounds, might be responsible for the significant antifungal activity of CF.

These results clearly demonstrated that the crude extracts and subfractions from the leaves of* Z. bungeanum* could be served as an accessible potential source for the production of functional food ingredients and medicinal exploration. This also could explain its frequent addition to the Chinese diet for promoting human health and for disease prevention. Further study is required to identify and quantify new bioactive compounds from EAF, AF, and CF fractions; major bioactive compounds especially are worthwhile to be isolated and purified. Also, further cellular and* in vivo* studies of their biological activities are required.

## Figures and Tables

**Table 1:** Content of total flavonoid, phenolic, and alkaloid and antioxidant capacity of ethanol extracts and its five fractions from *Z. bungeanum *leaves.

Samples	Content			Antioxidant capacity		
	TFC (mmol QE/100 g)	TPC (mmol GAE/100 g)	TAC (mmol CE/100 g)	DPPH_IC50 _(*μ*g/mL)	FRAP (*μ*mol Trolox/g)	ABTS (*μ*mol Trolox/g)
ECE	60.96 ± 8.87^a^	58.78 ± 9.37^b^	0.47 ± 0.28^c^	40.75 ± 0.21^c^	317.11 ± 9.71^b^	1122.91 ± 34.62^c^
PEF	4.01 ± 3.42^d^	4.43 ± 2.17^c^	1.71 ± 0.07^b^	377.95 ± 39.39^f^	81.56 ± 7.41^e^	264.20 ± 37.27^f^
CF	2.29 ± 2.03^d^	7.07 ± 2.36^c^	4.39 ± 0.05^a^	169.15 ± 4.60^e^	170.44 ± 10.45^d^	563.86 ± 22.66^e^
EAF	67.93 ± 9.29^a^	97.29 ± 17.53^a^	0.25 ± 0.02^c^	13.20 ± 0.85^b^	615.88 ± 1.86^b^	2147.83 ± 23.08^b^
AF	47.62 ± 6.34^bc^	62.87 ± 10.80^b^	0.06 ± 0.01^c^	18.55 ± 0.35^b^	594.15 ± 8.89^b^	2044.58 ± 19.99^b^
MF	31.63 ± 5.30^c^	8.35 ± 0.30^c^	0.10 ± 0^c^	85.85 ± 2.19^d^	191.93 ± 2.22^d^	747.69 ± 38.77^d^
Quercetin	—	—	—	2.60 ± 0.10^a^	1865.80 ± 33.40^a^	20113.58 ± 23.20^a^
Rutin	—	—	—	10.42 ± 0.14^ab^	1722.59 ± 6.42^a^	20153.46 ± 46.05^a^

TFC expressed as mmol quercetin equivalent per 100 g. TPC expressed as mmol gallic acid equivalent per 100 g. TAC expressed as mmol chelerythrine equivalent per 100 g. DPPH_IC50_ values were the effective concentrations at which DPPH radicals were scavenged by 50%. FRAP and ABTS results expressed as micromoles of trolox equivalent per g. Values are the mean of three replicates ± SD. Means with different letters within a column were significantly different (*P* < 0.05).

**Table 2:** Validation for the quantitative determination of twelve standard compounds using RP-HPLC.

Peak no.	Compound	Test range (*μ*g/mL)	Regression equation	Precision experiment		Repeatability		Recovery experiment	
				Area of peak (mAU∗min)	RSD (%)	Area of peak (mAU∗min)	RSD (%)	Average recovery rate (%)	RSD (%)
1	Chlorogenic acid	1~100	*y* = 7.747*x* − 1.0908 (*R* ^2^ = 0.9998)	377.97 ± 8.62	1.50	150.20 ± 1.65	2.38	99.87 ± 0.35	0.35
2	Epicatechin	10~500	*y* = 1.878*x* − 1.3776 (*R* ^2^ = 0.9999)	160.80 ± 2.41	1.11	265.33 ± 3.32	1.65	99.38 ± 1.65	1.66
3	Rutin	10~500	*y* = 22.356*x* + 55.311 (*R* ^2^ = 0.9999)	1042.93 ± 7.69	1.07	1944.37 ± 21.67	0.98	102.13 ± 0.88	0.86
4	Hyperoside	50~1000	*y* = 41.138*x* − 436.10 (*R* ^2^ = 0.9998)	865.77 ± 9.38	0.76	3573.98 ± 36.75	1.03	98.84 ± 0.93	0.94
5	Trifolin	1~50	*y* = 49.53*x* − 0.2025 (*R* ^2^ = 0.9999)	1000.50 ± 7.06	0.71	1125.80 ± 7.01	0.81	100.81 ± 1.02	1.01
6	Quercitrin	10~500	*y* = 63.459*x* − 171.41 (*R* ^2^ = 0.9994)	3641.90 ± 27.59	1.08	5210.20 ± 11.06	0.16	99.90 ± 0.31	0.31
7	Kaempferol-3-rhamnoside	1~100	*y* = 41.826*x* + 1.308 (*R* ^2^ = 0.9999)	2350.82 ± 35.73	0.74	753.97 ± 10.68	1.42	100.60 ± 0.91	0.91
8	Quercetin	1~50	*y* = 14.392*x* + 15.374 (*R* ^2^ = 0.9997)	182.53 ± 6.35	1.06	100.10 ± 4.83	0.92	101.35 ± 0.89	0.88
9	Nitidine chloride	0.1~5	*y* = 166.49*x* + 33.656 (*R* ^2^ = 0.9993)	140.71 ± 6.47	1.17	130.11 ± 5.52	1.11	99.75 ± 1.02	1.02
10	Chelerythrine	0.1~5	*y* = 175.41*x* + 0.7376 (*R* ^2^ = 0.9993)	114.54 ± 7.31	1.51	85.16 ± 6.22	1.56	103.76 ± 1.51	1.45
11	Xanthyletin	0.1~5	*y* = 98.498*x* + 67.619 (*R* ^2^ = 0.9995)	110.68 ± 8.35	1.12	93.52 ± 13.07	1.03	98.37 ± 1.04	1.06
15	Sesamin	10~500	*y* = 4.1445*x* + 107.11 (*R* ^2^ = 0.9991)	223.05 ± 8.03	1.32	215.04 ± 0.81	2.94	101.52 ± 1.32	1.75

Values were expressed as mean ± SD (*n* = 6).

**Table 3:** Content of twelve compounds in extracts and five fractions from *Z. bungeanum *leaves.

Peak no.	Compounds	Content (mg/g)					
		ECE	PEF	CF	EAF	AF	MF
1	Chlorogenic acid	3.78 ± 0.12^b^	ND	ND	2.96 ± 0.06^b^	5.87 ± 0.10^a^	2.40 ± 0.11^d^
2	Epicatechin	27.45 ± 0.93^c^	ND	ND	33.12 ± 0.61^b^	27.42 ± 0.17^c^	39.32 ± 1.30^a^
3	Rutin	16.86 ± 0.26^b^	ND	ND	4.79 ± 0.03^d^	29.94 ± 0.01^a^	14.72 ± 0.09^c^
4	Hyperoside	19.25 ± 0.55^c^	ND	ND	24.17 ± 0.90^b^	98.33 ± 1.14^a^	11.97 ± 0.10^d^
5	Trifolin	4.53 ± 0.07^c^	ND	ND	21.22 ± 0.12^b^	31.24 ± 0.78^a^	1.03 ± 0.01^d^
6	Quercitrin	16.73 ± 0.97^c^	ND	ND	125.21 ± 0.90^a^	116.63 ± 1.42^b^	2.49 ± 0.09^d^
7	Kaempferol-3-rhamnoside	3.75 ± 0.02^c^	0.48 ± 0.01^e^	1.23 ± 0.02^d^	54.95 ± 0.95^a^	16.71 ± 0.49^b^	ND
8	Quercetin	0.76 ± 0.03^b^	ND	ND	24.36 ± 0.71^a^	0.90 ± 0.05^b^	ND
9	Nitidine chloride	0.18 ± 0.003^a^	ND	ND	0.20 ± 0.004^a^	0.06 ± 0.003^b^	ND
10	Chelerythrine	0.09 ± 0.002^a^	ND	ND	ND	0.08 ± 0.005^a^	0.09 ± 0.003^a^
11	Xanthyletin	0.06 ± 0.01^b^	0.08 ± 0.01^b^	0.09 ± 0.01^a^	0.07 ± 0.005^c^	ND	ND
15	Sesamin	5.13 ± 0.11^c^	1.20 ± 0.14^d^	8.09 ± 1.07^b^	26.24 ± 0.87^a^	ND	ND

Values are the mean of three replicates ± SD. Means with different letters within a row were significantly different (*P* < 0.05). ND: not detectable.

**Table 4:** Correlation matrix between the results of the total phenolic and flavonoid content and the FRAP, ABTS, and DPPH activities.

	log DPPH	DPPH	ABTS	FRAP	TPC	TFC
log DPPH	1					
DPPH	−0.909∗	1				
ABTS	−0.968∗∗	−0.797	1			
FRAP	−0.958∗∗	−0.777	0.999∗∗	1		
TPC	−0.916∗	−0.829∗	0.827∗	0.808	1	
TFC	−0.922∗∗	−0.724	0.924∗∗	0.923∗∗	0.908∗	1

*Correlation is significant at the 0.05 level; **correlation is significant at the 0.01 level.

**Table 5:** Preliminary antifungal activity of ethanol extracts and its five fractions from *Z. bungeanum* leaves tested at 50 mg/mL against 20 plant pathogenic fungi.

Species	Inhibitory activity (%)					
	ECE	PEF	CF	EAF	AF	MF
*Alternaria alternata *	30.91 ± 1.57^c^	28.18 ± 1.57^c^	34.55 ± 0.00^b^	43.64 ± 1.57^a^	36.36 ± 3.15^b^	17.27 ± 1.57^d^
*Alternaria brassicae *	65.22 ± 3.77^ab^	60.87 ± 5.65^bc^	69.57 ± 1.88^a^	63.04 ± 3.77^abc^	57.61 ± 3.26^c^	60.87 ± 3.26^bc^
*Alternaria solani *	29.51 ± 1.42^d^	34.43 ± 1.42^c^	41.80 ± 1.42^b^	46.72 ± 1.42^a^	40.98 ± 2.46^b^	23.77 ± 2.46^e^
*Bipolaris sorokiniana *	24.55 ± 1.57^c^	27.27 ± 4.17^c^	35.45 ± 1.57^b^	50.91 ± 2.73^a^	28.18 ± 1.57^c^	24.55 ± 3.15^c^
*Botrytis cinerea *	38.76 ± 1.34^cd^	42.64 ± 2.69^c^	60.47 ± 2.33^a^	62.79 ± 2.33^a^	49.61 ± 5.85^b^	34.88 ± 6.15^d^
*Cladosporium fulvum *	29.03 ± 5.59^c^	41.94 ± 2.42^b^	50.81 ± 1.40^a^	50.81 ± 1.40^a^	43.55 ± 1.40^b^	25.00 ± 0.00^c^
*Colletotrichum gloeosporioides *	28.18 ± 4.17^c^	29.09 ± 5.45^c^	37.27 ± 2.73^b^	52.73 ± 4.17^a^	29.09 ± 2.73^c^	16.36 ± 1.57^d^
*Cucumis dahlia*	25.47 ± 1.63^c^	35.85 ± 1.63^b^	43.40 ± 2.83^a^	34.91 ± 2.83^b^	34.91 ± 2.83^b^	23.58 ± 2.83^c^
*Dothiorella gregaria *	45.38 ± 1.33^d^	52.31 ± 1.33^c^	70.00 ± 2.31^a^	62.31 ± 3.53^b^	46.15 ± 1.33^d^	44.62 ± 6.11^d^
*Fusarium oxysporum *	36.84 ± 2.63^c^	25.44 ± 1.52^d^	35.09 ± 1.52^c^	58.77 ± 3.04^a^	42.11 ± 0.11^b^	27.19 ± 1.52^d^
*Glomerella cingnlata *	50.00 ± 2.63^c^	53.51 ± 5.48^bc^	55.26 ± 0.00^b^	64.04 ± 1.52^a^	49.12 ± 1.52^c^	37.72 ± 1.52^d^
*Phacidiopycnis washingtonensis *	24.14 ± 2.99^c^	25.00 ± 2.59^c^	35.34 ± 2.59^b^	50.00 ± 1.49^a^	37.07 ± 1.49^b^	35.34 ± 2.59^b^
*Physalospora piricola *	43.75 ± 8.33^bc^	45.83 ± 5.51^bc^	51.39 ± 1.20^b^	60.42 ± 2.08^a^	51.39 ± 3.18^b^	40.97 ± 1.20^c^
*Piricularia oryzae *	42.22 ± 4.44^d^	51.85 ± 2.57^c^	68.15 ± 1.28^a^	60.74 ± 1.28^b^	49.63 ± 1.28^c^	43.70 ± 1.28^d^
*Rhizoctonia cerealis *	6.00 ± 3.46^b^	10.00 ± 6.00^b^	28.00 ± 6.00^a^	34.00 ± 6.00^a^	24.00 ± 3.46^a^	26.00 ± 9.17^a^
*Sclerotinia sclerotiorum *	19.89 ± 1.70^d^	56.82 ± 2.60^b^	59.66 ± 2.60^ab^	64.20 ± 1.70^a^	29.55 ± 4.92^c^	26.70 ± 2.95^c^
*Thanatephorus cucumeris *	31.03 ± 3.95^d^	37.93 ± 2.59^bc^	34.48 ± 3.95^bcd^	40.52 ± 4.48^a^	33.62 ± 2.99^cd^	32.76 ± 2.59^cd^
*Valsa mali *	23.64 ± 2.73^c^	26.36 ± 2.73^c^	42.73 ± 2.73^b^	50.91 ± 2.73^a^	39.09 ± 4.17^b^	15.45 ± 2.73^d^
*Venturia pyrina *	54.62 ± 2.66^a^	47.69 ± 1.33^b^	56.92 ± 3.53^a^	58.46 ± 0.00^a^	54.62 ± 1.33^a^	37.69 ± 2.31^c^
*Verticillium dahliae *	24.07 ± 3.21^b^	21.30 ± 5.78^b^	35.19 ± 1.60^a^	37.96 ± 3.21^a^	34.26 ± 1.60^a^	23.15 ± 1.60^b^

Values are the mean of three replicates ± SD. Means with different letters within a row were significantly different (*P* < 0.05).

**Table 6:** EC_50_ values of ethanol extracts and its five fractions from *Z. bungeanum *leaves against 5 selected plant pathogenic fungi.

Sample	EC_50_ (mg/mL)				
	*Botrytis cinerea *	*Piricularia oryzae *	*Physalospora piricola *	*Glomerella cingulata *	*Venturia pyrina *
ECE	11.82 ± 1.15^ab^	12.31 ± 0.45^a^	39.48 ± 2.25^ab^	13.00 ± 1.34^bc^	33.22 ± 3.61^d^
PEF	69.34 ± 3.99^d^	30.02 ± 3.25^ab^	65.32 ± 2.39^b^	32.83 ± 4.61^d^	31.77 ± 0.77^cd^
CF	9.39 ± 0.17^ab^	4.18 ± 0.08^a^	10.89 ± 1.62^ab^	0.83 ± 0.24^a^	5.35 ± 0.34^ab^
EAF	24.39 ± 2.38^b^	17.81 ± 0.19^a^	13.73 ± 0.69^ab^	9.25 ± 0.11^b^	8.11 ± 0.74^b^
AF	51.16 ± 3.54^c^	75.63 ± 18.53^b^	46.69 ± 11.08^ab^	14.96 ± 1.11^c^	26.44 ± 3.11^c^
MF	598.31 ± 18.91^e^	625.81 ± 49.58^c^	646.04 ± 56.20^c^	227.90 ± 2.64^e^	110.18 ± 4.15^e^
Amphotericin	0.03^a^	0.01^a^	0.08^a^	0.01^a^	0.37^a^

Values are the mean of three replicates ± SD. Means with different letters within a column were significantly different (*P* < 0.05).

**Table 7:** MIC values of ethanol extracts and its five fractions from *Z. bungeanum *leaves against 3 selected bacteria.

Bacteria	MIC (mg/mL)						
	ECE	PEF	CF	EAF	AF	MF	Benzylpenicillin
*Staphylococcus aureus *	4.93 ± 0.69^c^	2.45 ± 1.25^b^	4.76 ± 0.17^c^	2.38 ± 0.82^b^	5.15 ± 0.16^c^	2.65 ± 0.97^b^	1.31 ± 0.01^a^
*Escherichia coli *	4.61 ± 2.11^bc^	4.34 ± 1.78^bc^	3.15 ± 0.72^ab^	2.32 ± 0.83^a^	5.70 ± 0.31^c^	3.10 ± 0.43^ab^	>10
*Bacillus subtilis *	8.29 ± 0.98^c^	4.78 ± 0.42^b^	4.48 ± 0.79^b^	4.24 ± 0.54^b^	5.42 ± 1.04^b^	4.10 ± 0.23^b^	0.01 ± 0^a^

Values are the mean of three replicates ± SD. Means with different letters within a row were significantly different (*P* < 0.05). Benzylpenicillin was used as the positive control.
